# Localization Framework for Real-Time UAV Autonomous Landing: An On-Ground Deployed Visual Approach

**DOI:** 10.3390/s17061437

**Published:** 2017-06-19

**Authors:** Weiwei Kong, Tianjiang Hu, Daibing Zhang, Lincheng Shen, Jianwei Zhang

**Affiliations:** 1 College of Mechatronics and Automation, National University of Defense Technology, Changsha 410073, China; kongww.nudt@gmail.com (W.K.); swimsword@163.com (D.Z.); lcshen@nudt.edu.cn (L.S.); 2 Naval Academy of Armament, Beijing 100161, China; 3 Institute of Technical Aspects of Multimodal Systems (TAMS), Department of Computer Science, University of Hamburg, 22527 Hamburg, Germany; zhang@informatik.uni-hamburg.de

**Keywords:** UAV, stereo vision, localization

## Abstract

One of the greatest challenges for fixed-wing unmanned aircraft vehicles (UAVs) is safe landing. Hereafter, an on-ground deployed visual approach is developed in this paper. This approach is definitely suitable for landing within the global navigation satellite system (GNSS)-denied environments. As for applications, the deployed guidance system makes full use of the ground computing resource and feedbacks the aircraft’s real-time localization to its on-board autopilot. Under such circumstances, a separate long baseline stereo architecture is proposed to possess an extendable baseline and wide-angle field of view (FOV) against the traditional fixed baseline schemes. Furthermore, accuracy evaluation of the new type of architecture is conducted by theoretical modeling and computational analysis. Dataset-driven experimental results demonstrate the feasibility and effectiveness of the developed approach.

## 1. Introduction

Over the past few decades, the application of unmanned aircraft has increased enormously in both civil and military scenarios. Although aerial robots have successfully been implemented in several applications, there are still new research directions related to them. Floreano [1] and Kumar et al. [2] outlined the opportunities and challenges of this developing field, from the model design to high-level perception capability. All of these issues are concentrating on improving the degree of autonomy, which supports that UAVs continue to be used in novel and surprising ways. No matter whether fixed-wing or rotor-way platforms, a standard fully-unmanned autonomous system (UAS) involves performs takeoffs, waypoint flight and landings. Among them, the landing maneuver is the most delicate and critical phase of UAV flights. Two technical reports [3] argued that nearly 70% of mishaps of Pioneer UAVs were encountered during the landing process caused by human factors. Therefore, a proper assist system is needed to enhance the reliability of the landing task. Generally, two main capabilities of the system are required. The first one is localization and navigation of UAVs, and the second one is generating the appropriate guidance command to guide UAVs for a safe landing.

For manned aircraft, the traditional landing system uses a radio beam directed upward from the ground [4,5]. By measuring the angular deviation from the beam through onboard equipment, the pilot knows the perpendicular displacement of the aircraft in the vertical channel. For the azimuth information, additional equipment is required. However, due to the size, weight and power (SWaP) constraints, it is impossible to equip these instruments in UAV. Thanks to the GNSS technology, we have seen many successful practical applications of autonomous UAVs in outdoor environments such as transportation, aerial photography and intelligent farming. Unfortunately, in some circumstances, such as urban or low altitude operations, the GNSS receiver antenna is prone to lose line-of-sight with satellites, making GNSS unable to deliver high quality position information [6]. Therefore, autonomous landing in an unknown or global navigation satellite system (GNSS)-denied environment is still an open problem.

The visual-based approach is an obvious way to achieve the autonomous landing by estimating flight speed and distance to the landing area, in a moment-to-moment fashion. Generally, two types of visual methods can be considered. The first category is the vision-based onboard system, which has been widely studied. The other is to guide the aircraft using a ground-based camera system. Once the aircraft is detected by the camera during the landing process, its characteristics, such as type, location, heading and velocity, can be derived by the guidance system. Based on this information, the UAV could align itself carefully towards the landing area and adapt its velocity and acceleration to achieve safe landing. In summary, two key elements of the landing problem are detecting the UAV and its motion, calculating the location of the UAV relative to the landing field.

**Figure 1:**
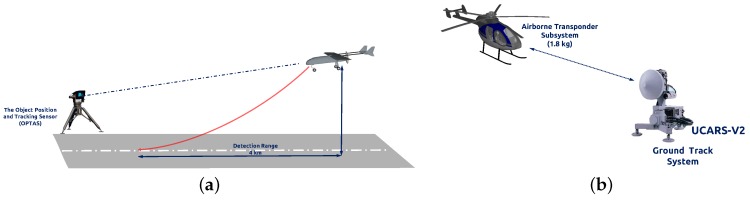
(**a**) The object position and tracking sensor (OPTAS) system is mounted on the tripod. Adapted from [7]; (**b**) UAS common automatic recovery system (UCARS) for MQ-8B autonomous landing. Adapted from [8].

To achieve better performance in GNSS-denied environments, some other types of sensors, such as laser range finders, millimeter wavelength radar, have been explored for UAV autonomous landing. Swiss company RUAG (Bern, Switzerland) solved the landing task by the OPATS (object position and tracking sensor) [7]. Figure 1a presents this laser-based automatic landing system, the infrared laser beam of which is echoed back from a passive and optionally heated retro reflector on the aircraft. This system could measure the position of approaching aircraft around 4000 m. Moreover, the Sierra Nevada Corporation provides an alternative to the laser-based method. They developed the UAS common automatic recovery system (UCARS) [8] based on millimeter wavelength ground radar for MQ-8B Fire Scout autonomous landing, as shown in Figure 1b. Benefiting from the short bandwidth, UCARS provides precision approach (within 2.5 cm) in adverse weather condition. While those solutions are effective, they require the use of radar or laser emissions, which can be undesirable in a tactical situation. Furthermore, the limited payload of a small UAV constrains the onboard modules.

Motivated by these mentioned challenges, we propose and develop a novel on-ground deployment of the visual landing system. In this paper, we mainly focus on the localization and navigation issue and try to improve the navigation accuracy and robustness. The essential contributions of this work are as follows: (1) an extendable baseline and wide-angle field of view (FOV) vision guidance system is developed by using a physically-separated and informationally-connected deployment of the two PTUs on both sides of the runway; (2) localization error and its transferring mechanism in practical situations are unveiled with both theoretical and computational analyses. In particular, the developed approach is experimentally validated with fair accuracy and better performance in timeliness, as well as practicality against the previous works.

The remainder of this paper is organized as follows. Section 2 briefly reviews the related works. In Section 3, the architecture of the on-ground deployed stereo system is proposed and designed. Section 4 conducts the accuracy evaluation, and its transferring mechanism is conducted through theoretical and computational analysis. Dataset-driven validation is followed in Section 5. Finally, concluding remarks are presented in Section 6.

## 2. Related Works

While several techniques have been applied for onboard vision-based control of UAVs, few have shown landing of a fixed-wing guiding by a ground-based system. In 2006, Wang [9] proposed a system using a step motor controlling a web camera to track and guide a micro-aircraft. This camera rotation platform expands the recognition area from 60 cm × 60 cm–140 cm × 140 cm, but the range of the recognition is only 1 m. This configuration cannot be used to determine the position of a fixed-wing in the field.

At Chiba University [10], a ground-based Bumblebee stereo vision system was used to calculate the 3D position of a quadrotor at the altitude of 6 m. The Bumblebee has a 15.7-cm baseline with a 66° horizontal field of view. The sensor was mounted on a tripod with the height of 45 cm, and the drawback of this system is the limited baseline leading to a narrow field of view (FOV).

To increase the camera FOV, multi-camera systems are considered attractive. This kind of system could solve the common vision problems and track objects to compute their 3D locations. In addition, Martinez [11] introduced a trinocular on-ground system, which is composed of three or more cameras for extracting key features of the UAV to obtain robust 3D position estimation. The lenses of the FireWire cameras are 3.4 mm and capture images of a 320×240 size at 30 fps. They employed the continuously-adaptive mean shift (CamShift) algorithm to track the four cooperation markers with independent color, which were distributed on the bottom of the helicopter. The precision of this system in the vertical and horizontal direction is around 5 cm and in depth estimation is 10 cm with a 3-m recognition range. The maximum range for depth estimation is still not sufficient for fixed-wing UAV. Additionally, another drawback of the multi-camera system is the calibration process, whose parameters are nontrivial to obtain.

A state-of-the-art study from Guan et al. [12] proposed a multi-camera network with laser rangefinders to estimate an aircraft’s motion. This system is composed of two sets of measurement units that are installed on both sides of the runway. Each unit has three high-speed cameras with different focal lengths and FOV to captures the target in the near-filed (20 m–100 m), middle-field (100 m–500 m) and far-field (500 m–1000 m), respectively. A series of field experiments shows that the RMS error of the distance is 1.32 m. Due to the configuration of the system, they have to apply a octocopter UAV equipped with a prism to calibrate the whole measurement system.

**Figure 2:**
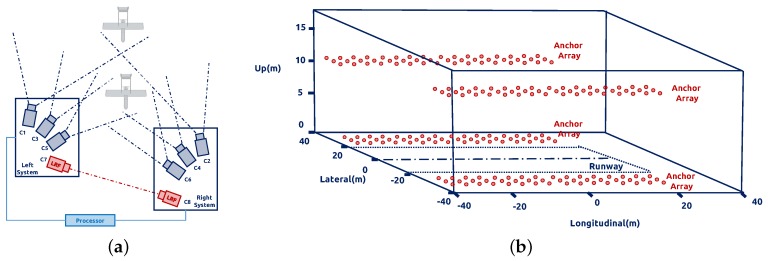
(**a**) Architecture of the multi-camera network. (**b**) In the longitudinal direction, there are 20 anchors separated by 3 m. In the vertical direction, the anchors are either located on the ground or at the 10-m height antennas. The red points show the 240 possible anchor locations.

Except the camera-based ground navigation system, the ultra-wide band (UWB) positioning network is also discussed in the community. Kim and Choi [13] deployed the passive UWB anchors by the runway, which listen for the UWB signals emitted from the UAV. The ground system computes the position of the target based on the geometry of the UWB anchors and sends it back to the UAV through the aviation communication channel. There are a total of 240 anchor possible locations, as shown in Figure 2b, distributed at each side of the runway, and the longitudinal range is up to 300 m with a positioning accuracy of 40 cm.

**Figure 3:**
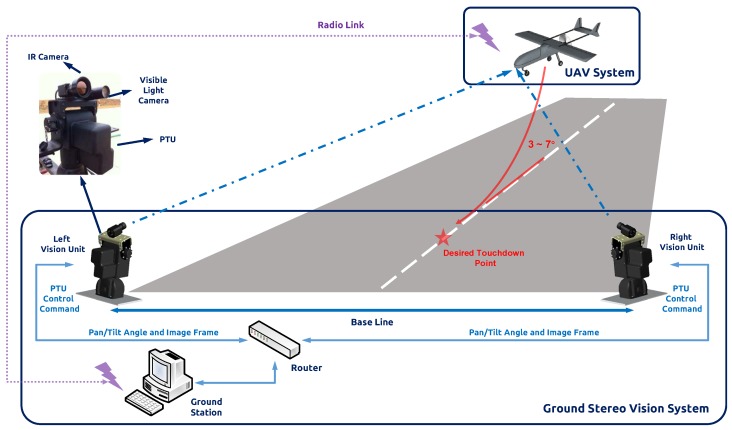
Architecture of the ground stereo vision system.

Our group first developed the traditional stereo ground-based system with infrared cameras [14], while this system has limited detection distance. For short-baseline configuration, cameras were setup on one PTU, and the system should be mounted on the center line of the runway. However, the short-baseline limits the maximum range for UAV depth estimation. To enhance the operating capability, we conducted the triangular geometry localization method for the PTU-based system [15]. As shown in Figure 3, we fixed the cameras with separate PTUs on the both sides of the runway. Therefore, the landing aircraft can be locked by our system around 1 km. According to the previous work, the localization accuracy largely depends on the aircraft detection precision in the camera image plane. Therefore, we implemented the Chan–Vese method [16] and the saliency-inspired method [17] to detect and track the vehicle more accurately; however, these approaches are not suitable for real-time requirements.

For more information, we also reviewed various vision-based landing approaches performed on different platforms [18], and Gautam provides another general review of the autonomous landing techniques for UAVs [19].

## 3. System Architecture and Deployment

In this section, we introduce the theoretical model for the ground-to-air visual system. We first recap the traditional stereo vision model, which has a limited baseline, restraining the detection distance. To enlarge the system working boundary, we setup the camera and other sensor modules on the two separated PTUs and then calculate the target according to the image information and rotation angle from PTU. Each vision unit works independently and transfers the results of image processing and PTU status to the navigation computer, which calculates the estimated relative position of the UAV. The architecture of the ground stereo vision system is shown in Figure 3.

### 3.1. Fundamental Principles of Ground-Based Stereo Systems

The standard camera model is a pin-hole camera model. The coordinate of the target *M* is (x,y,z), and its position on the image plane is (u,v). The camera focus is *f*; then, the relationship of the coordinate between the 3D world and 2D image plane can be calculated by:

(1) $$\lambda \left[\begin{array}{c} u\\ v\\ f \end{array}\right]=\left[\begin{array}{c} x\\ y\\ z \end{array}\right]$$

where λ is the scale factor.

Although the above model is simple, it could be helpful to estimate the theoretical camera lens according to the expected distance and resolution or to measure the target size roughly based on the pixel length on the image plane. Let the width and height of the target be *W* and *H*; the distance between the camera and target be *L*; the target projection on image plane be *w* and *h*; the relationship between them is:

(2) $$\begin{array}{c} f=\frac{wL}{W}\;f=\frac{hL}{H} \end{array}$$

**Figure 4:**
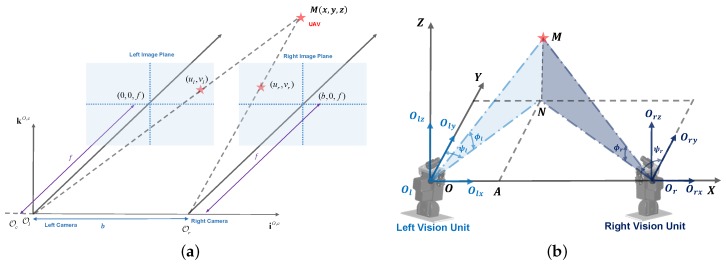
(**a**) Theoretical stereo vision model; (**b**) theoretical optical model. The target *M* is projected at the center of the optical axis.

We define the coordinates of the left and right navigation module as shown in Figure 4b. When the optical axes of these two cameras are parallel, we could calculate the target in 3D space by:

(3) $$\left[\begin{array}{c} x\\ y\\ z \end{array}\right]=\frac{b}{d}\left[\begin{array}{c} u_{l}\\ u_{r}\\ f \end{array}\right]$$

where *b* is the baseline and $d=u_{l}-u_{r}$ is the pixel disparity, as shown in Figure 4a. Even though some calibration methods could manage the axes’ nonparallel situation, it is still difficult to calculate the system correctly, as the baseline is large.

### 3.2. Separated Long Baseline Deployment

In order to detect the target at long distance, a large baseline, more than 5 m, is required. Benefiting the camera assembled on the PTU separately, we could switch the baseline freely according to the expected detection distance and target size.

In this paper, we assumed that the world coordinate system (X,Y,Z) is located on the origin of the left vision unit, the rotation center of the PTU. For the sake of simplicity, the camera is installed on the PTU in the way that the axes of the camera frame are parallel to those of the PTU frame. The origins of these two frames are close. Therefore, it can be assumed that the camera frame coincides with the body frame. Figure 4b reveals the theoretical model for visual measurement. After installing the right camera system on the *X*-axis, the left and right optical center can be expressed as $O_{l}$ and $O_{r}$, respectively. Then, the baseline of the optical system is $O_{l}O_{r}$, whose distance is *D*. Considering the center of mass of the UAV as a point *M*, $O_{l}M$ and $O_{r}M$ illustrate the connections between the each optical center and the UAV. In addition, $\phi_{l}$, $\phi_{r}$, $\psi_{l}$, $\psi_{r}$ denote the tilt and pan angle on both sides. Therefore, we define $\phi_{l}=0$, $\phi_{r}=0$, $\psi_{l}=0$ and $\psi_{r}=0$, as the PTU is set to the initial state, i.e., the optical axis parallel to the runway; the measurement of the counterclockwise direction is positive.

**Figure 5:**
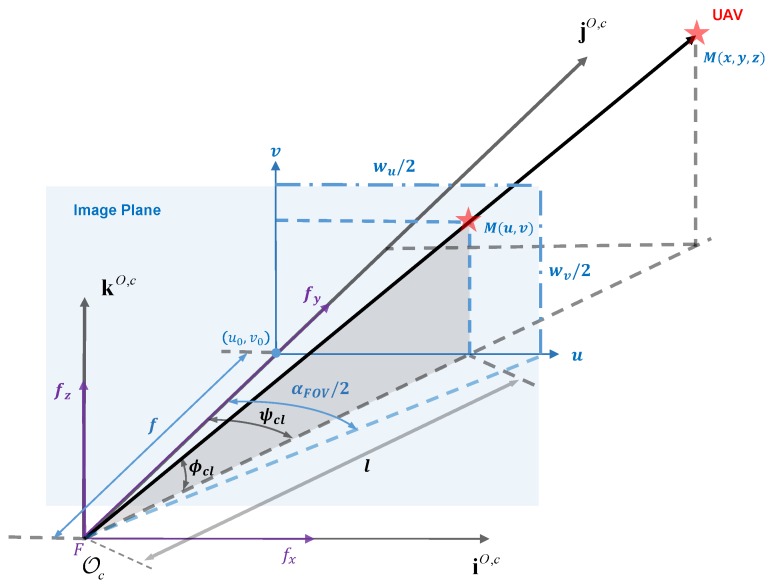
The geometry of one PTUwith respect to the optical center and the image plane.

Since the point *M* does not coincide with the principle point, which is the center of the image plane, the pixel deviation compensation in the longitudinal and horizontal direction should be considered. As shown in Figure 5, we calculate pixel deviation compensation on the left side by:

(4) $$\left\{\begin{array}{c} \psi_{cl}=\arctan\frac{(u-u_{0})du}{f}\\ \phi_{cl}=\arctan\frac{\left(v-v_{0}\right)\cos\psi_{cl}dv}{f} \end{array}\right.$$

where the optical point is $o(u_{o},v_{o})$, du and dv are the pixel length of the *u*- and *v*-axis in image plane and *f* is the focus. The current PTU rotation angle can be directly obtained through the serial ports during the experiments. Let $\phi_{pl}$ and $\psi_{pl}$ be the left pan and tilt angle separately. Then, the total pan and tilt angle on the left side can be detailed as:

(5) $$\left\{\begin{array}{cc} \phi_{l} & =\phi_{cl}+\phi_{pl}\\ \psi_{l} & =\psi_{cr}+\psi_{pr} \end{array}\right.$$

For the other side, we could also calculate the angle in the same way.

The world coordinates of point *M* is $\left(x_{M},y_{M},z_{M}\right)\in R^{3}$. Point *N* is the vertical projection of point *M* on the XOY plane, and NA is perpendicular to the *X*-axis. If we define NA=h, the following navigation parameters can be obtained:

(6) $$\left\{\begin{array}{c} x_{M}=h\tan\psi_{l}=\frac{D\tan\psi_{l}}{\tan\psi_{l}-\tan\psi_{r}}\\ y_{M}=h=\frac{D}{\tan\psi_{l}-\tan\psi_{r}}\\ z_{M}=\frac{h\tan\phi_{l}}{\cos\psi_{l}}=\frac{D\tan\phi_{l}}{\cos\psi_{l}\left(\tan\psi_{l}-\tan\psi_{r}\right)} \end{array}\right.$$

Furthermore, errors in the internal and external camera calibration parameters marginally affect some of the estimates: the *x*-position and *z*-position, in particular.

## 4. Accuracy Evaluation of the On-Ground Stereo System

### 4.1. Theoretical Modeling

We are now in the position to analyze the error related to the PTU rotation angle. The discussion was first presented in our previous works [15]. According to Equation (6), the partial derivatives of each equation with respect to the pan angle and the tilt angle are denoted in the following way,

(7) $$\left\{\;\begin{array}{c} \frac{\partial x_{M}}{\partial \psi_{l}}=\frac{D\tan\psi_{r}}{\cos^{2}\psi_{l}{\left(\tan\psi_{l}-\tan\psi_{r}\right)}^{2}}\\ \frac{\partial x_{M}}{\partial \psi_{r}}=\frac{D\tan\psi_{l}}{\cos^{2}\psi_{r}{\left(\tan\psi_{l}-\tan\psi_{r}\right)}^{2}} \end{array}\right.$$

(8) $$\left\{\;\begin{array}{c} \frac{\partial y_{M}}{\partial \psi_{l}}=\frac{D}{\cos^{2}\psi_{l}{\left(\tan\psi_{l}-\tan\psi_{r}\right)}^{2}}\\ \frac{\partial y_{M}}{\partial \psi_{r}}=\frac{D}{\cos^{2}\psi_{r}{\left(\tan\psi_{l}-\tan\psi_{r}\right)}^{2}} \end{array}\right.$$

(9) $$\left\{\;\begin{array}{c} \frac{\partial z_{M}}{\partial \phi_{l}}=\frac{D}{\cos\psi_{l}\cos^{2}\phi_{l}\left(\tan\psi_{l}-\tan\psi_{r}\right)}\\ \frac{\partial z_{M}}{\partial \psi_{l}}=\frac{D\tan\phi_{l}\left(\cos\psi_{l}+\sin\psi_{l}\tan\psi_{r}\right)}{\cos^{2}\psi_{r}{\left(\tan\psi_{l}-\tan\psi_{r}\right)}^{2}}\\ \frac{\partial z_{M}}{\partial \psi_{r}}=\frac{D\tan\phi_{l}}{\cos\psi_{l}\cos^{2}\psi_{r}{\left(\tan\psi_{l}-\tan\psi_{r}\right)}^{2}} \end{array}\right.$$

To analyze the influence of the error from the angle, we define the gradient of the world coordinate as:

(10) $$\nabla_{x_{M}}\left(\psi_{l},\psi_{r}\right):=\left(\frac{\partial x_{M}}{\partial \psi_{l}}\left(\psi_{l},\psi_{r}\right),\frac{\partial x_{M}}{\partial \psi_{r}}\left(\psi_{l},\psi_{r}\right)\right)$$

(11) $$\nabla_{y_{M}}\left(\psi_{l},\psi_{r}\right):=\left(\frac{\partial y_{M}}{\partial \psi_{l}}\left(\psi_{l},\psi_{r}\right),\frac{\partial y_{M}}{\partial \psi_{r}}\left(\psi_{l},\psi_{r}\right)\right)$$

(12) $$\nabla_{z_{M}}\left(\psi_{l},\psi_{r}\right):=\left(\frac{\partial z_{M}}{\partial \psi_{l}}\left(\psi_{l},\psi_{r}\right),\frac{\partial z_{M}}{\partial \psi_{r}}\left(\psi_{l},\psi_{r}\right)\right)$$

**Figure 6:**
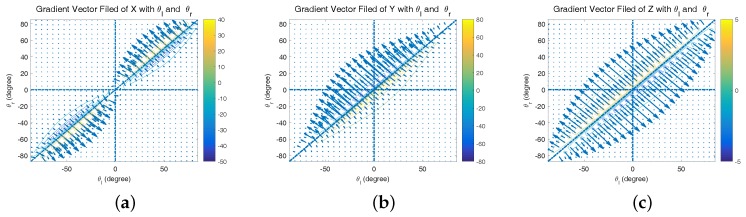
Vector field distribution of (**a**) $\nabla_{x_{M}}\left(\psi_{l},\psi_{r}\right)$, (**b**) $\nabla_{y_{M}}\left(\psi_{l},\psi_{r}\right)$ and (**c**) $\nabla_{z_{M}}\left(\psi_{l},\psi_{r}\right)$.

**Figure 7:**
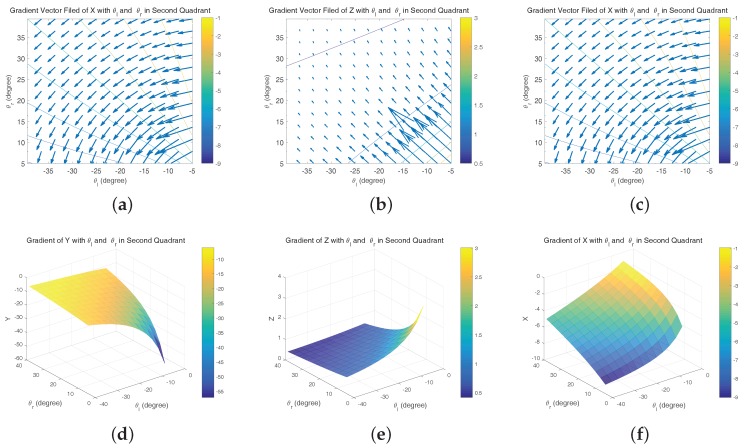
(**a**–**c**) Vector field distribution of $\nabla_{x_{M}}\left(\psi_{l},\psi_{r}\right)$, $\nabla_{y_{M}}\left(\psi_{l},\psi_{r}\right)$, and $\nabla_{z_{M}}\left(\psi_{l},\psi_{r}\right)$ in the second quadrant; (**d**–**f**) gradient of *X*, *Y*, *Z* with $\theta_{l}$ and $\theta_{r}$ in the second quadrant.

In this case, simulation is needed to evaluate the behavior of our visual system. Figure 6a–c is the vector field distribution of $\nabla_{x_{M}}\left(\psi_{l},\psi_{r}\right)$, $\nabla_{y_{M}}\left(\psi_{l},\psi_{r}\right)$ and $\nabla_{z_{M}}\left(\psi_{l},\psi_{r}\right)$, which give us an intuitive result under different types of errors. The length of each vector describes the strength at a specific point; the direction along the vector points to the direction of the fastest error increase. However, only when $y_{M}\geq 0$ (the aircraft is in front of two cameras), the area $\psi_{l}-\psi_{r}>0$ has the physics meaning. Figure 7a shows that $x_{M}$ has a significant variation when $\psi_{l}$ is approximate to $\psi_{r}$, namely the optical axes are nearly parallel. Further, $y_{M}$ and $z_{M}$ have similar variations. Considering the general working status of the ground-based system, we mainly focus on the second quadrant of the aforementioned vector fields as shown in Figure 7a–c. In these areas, there are slight variations that theoretically demonstrate the feasibility of the system.

### 4.2. Computational Analysis

In theory, $O_{l}M$ and $O_{r}M$ should intersect perfectly at one point all of the time, as shown in Figure 4b. Due to the inevitable errors from PTU rotation and tracking algorithms, we estimate the intersecting point by combing the vertical line of two different planes in space.

(1) We set $\left(x_{ol},y_{ol},z_{ol}\right)=\left(0,0,0\right)$, and $\left(x_{or},y_{or},z_{or}\right)=\left(D,0,0\right)$ is the optical center of each camera. Assuming that $a_{l}\neq 0$, $b_{l}\neq 0$, $c_{l}\neq 0$ and $a_{r}\neq 0$, $b_{r}\neq 0$, $c_{r}\neq 0$, we obtain the parametric equations of lines $O_{l}M$ and $O_{r}M$:

(13) $$\left\{\begin{array}{c} \frac{x-x_{ol}}{a_{l}}=\frac{y-y_{ol}}{b_{l}}=\frac{z-z_{ol}}{c_{l}}=t_{l},\\ \frac{x-x_{or}}{a_{r}}=\frac{y-y_{or}}{b_{r}}=\frac{z-z_{or}}{c_{r}}=t_{r}, \end{array}\right.$$

(14) $$\left\{\begin{array}{c} a_{l}=\cos\phi_{l}\sin\psi_{l}\\ b_{l}=\cos\phi_{l}\cos\psi_{l}\\ c_{l}=\sin\phi_{l} \end{array}\right.\;\left\{\begin{array}{c} a_{r}=\cos\phi_{r}\sin\psi_{r}\\ b_{r}=\cos\phi_{r}\cos\psi_{r}\\ c_{r}=\sin\phi_{r} \end{array}\right.$$

where $t_{l}$, $t_{r}$ are the parameters for the line $O_{l}M$ and $O_{r}M$ separately. Therefore, any point (x,y,z) on each line is usually written parametrically as a function of $t_{l}$ and $t_{r}$:

(15) $$\left\{\begin{array}{c} x_{l}=a_{l}t_{l}+x_{ol}\\ y_{l}=b_{l}t_{l}+y_{ol}\\ z_{l}=c_{l}t_{l}+z_{ol} \end{array}\right.\;\left\{\begin{array}{c} x_{r}=a_{r}l_{r}+x_{or}\\ y_{r}=b_{r}t_{r}+y_{or}\\ z_{r}=c_{r}t_{r}+z_{or} \end{array}\right.$$

(2) In our situation, $O_{l}M$ and $O_{r}M$ are skew lines, such that these two lines are no parallel and do not intersect in 3D. Generally, the shortest distance between the two skew lines lies along the line that is perpendicular to both of them. By defining the intersection points of the shortest segment line for each line by $\left(x_{lp},y_{lp},z_{lp}\right)$ and $\left(x_{rp},y_{rp},z_{rp}\right)$, we get the parametric equations:

(16) $$\left\{\begin{array}{c} x_{lp}=a_{l}t_{l}+x_{ol}\\ y_{lp}=b_{l}t_{l}+y_{ol}\\ z_{lp}=c_{l}t_{l}+z_{ol} \end{array}\right.\;\left\{\begin{array}{c} x_{rp}=a_{r}l_{r}+x_{or}\\ y_{rp}=b_{r}t_{r}+y_{or}\\ z_{rp}=c_{r}t_{r}+z_{or} \end{array}\right.$$

(3) Knowing the position of the intersection points on each line, the distance is calculated by the square Euclidean norm:

(17) $$J={∥\left(x_{lp},y_{lp},z_{lp}\right)-(x_{rp},y_{rp},z_{rp})∥}_{2}^{2}$$

(4) By deriving the function *J*, we achieved the minimum distance when $\frac{\partial J}{\partial t_{l}}=0$ and $\frac{\partial J}{\partial t_{r}}=0$. Then, the above functions derive the following equation:

(18) $$\begin{array}{c} \left[\begin{array}{cc} a_{l}^{2}+b_{l}^{2}+c_{l}^{2} & -(a_{l}a_{r}+b_{l}b_{r}+c_{l}c_{r})\\ -(a_{l}a_{r}+b_{l}b_{r}+c_{l}c_{r}) & a_{l}^{2}+b_{l}^{2}+c_{l}^{2} \end{array}\right]\left[\begin{array}{c} t_{l}\\ t_{r} \end{array}\right]\\ =\left(x_{ol}-x_{or}\right)\left[\begin{array}{c} -a_{l}\\ a_{r} \end{array}\right]+\left(y_{ol}-y_{or}\right)\left[\begin{array}{c} -b_{l}\\ b_{r} \end{array}\right]+\left(z_{ol}-z_{or}\right)\left[\begin{array}{c} -c_{l}\\ c_{r} \end{array}\right]. \end{array}$$

We could define the matrix on the left side as:

(19) $$\begin{array}{c} H=\left[\begin{array}{cc} a_{l}^{2}+b_{l}^{2}+c_{l}^{2} & -(a_{l}a_{r}+b_{l}b_{r}+c_{l}c_{r})\\ -(a_{l}a_{r}+b_{l}b_{r}+c_{l}c_{r}) & a_{l}^{2}+b_{l}^{2}+c_{l}^{2} \end{array}\right] \end{array}$$

Considering that there is a uniqueness vertical line, so detH≠0, and the position of the target point Min the world coordinate is:

(20) $$\begin{array}{c} \left[\begin{array}{c} x_{M}\\ y_{M}\\ z_{M} \end{array}\right]=w\left[\begin{array}{c} x_{lp}\\ y_{lp}\\ z_{lp} \end{array}\right]+\left(1-w\right)\left[\begin{array}{c} x_{rp}\\ y_{rp}\\ z_{rp} \end{array}\right],w\in \left[0,1\right]. \end{array}$$

where *w* is weight, and the other parameters are:

(21) $$\left\{\begin{array}{c} x_{lp}=a_{l}D\frac{a_{l}\left(a_{l}^{2}+b_{l}^{2}+c_{l}^{2}\right)-a_{r}\left(a_{l}a_{r}+b_{l}b_{r}+c_{l}c_{r}\right)}{{\left(a_{l}b_{r}-b_{l}a_{r}\right)}^{2}+{\left(b_{l}c_{r}-c_{l}b_{r}\right)}^{2}+{\left(a_{l}c_{r}-c_{l}a_{r}\right)}^{2}}\\ y_{lp}=b_{l}D\frac{a_{l}\left(a_{l}^{2}+b_{l}^{2}+c_{l}^{2}\right)-a_{r}\left(a_{l}a_{r}+b_{l}b_{r}+c_{l}c_{r}\right)}{{\left(a_{l}b_{r}-b_{l}a_{r}\right)}^{2}+{\left(b_{l}c_{r}-c_{l}b_{r}\right)}^{2}+{\left(a_{l}c_{r}-c_{l}a_{r}\right)}^{2}}\\ z_{lp}=c_{l}D\frac{a_{l}\left(a_{l}^{2}+b_{l}^{2}+c_{l}^{2}\right)-a_{r}\left(a_{l}a_{r}+b_{l}b_{r}+c_{l}c_{r}\right)}{{\left(a_{l}b_{r}-b_{l}a_{r}\right)}^{2}+{\left(b_{l}c_{r}-c_{l}b_{r}\right)}^{2}+{\left(a_{l}c_{r}-c_{l}a_{r}\right)}^{2}} \end{array}\right.$$

and:

(22) $$\left\{\begin{array}{c} x_{rp}=D\left[a_{r}\frac{a_{l}\left(a_{l}a_{r}+b_{l}b_{r}+c_{l}c_{r}\right)-a_{r}\left(a_{l}^{2}+b_{l}^{2}+c_{l}^{2}\right)}{{\left(a_{l}b_{r}-b_{l}a_{r}\right)}^{2}+{\left(b_{l}c_{r}-c_{l}b_{r}\right)}^{2}+{\left(a_{l}c_{r}-c_{l}a_{r}\right)}^{2}}+1\right]\\ y_{rp}=b_{r}D\frac{a_{l}\left(a_{l}a_{r}+b_{l}b_{r}+c_{l}c_{r}\right)-a_{r}\left(a_{l}^{2}+b_{l}^{2}+c_{l}^{2}\right)}{{\left(a_{l}b_{r}-b_{l}a_{r}\right)}^{2}+{\left(b_{l}c_{r}-c_{l}b_{r}\right)}^{2}+{\left(a_{l}c_{r}-c_{l}a_{r}\right)}^{2}}\\ z_{rp}=c_{r}D\frac{a_{l}\left(a_{l}a_{r}+b_{l}b_{r}+c_{l}c_{r}\right)-a_{r}\left(a_{l}^{2}+b_{l}^{2}+c_{l}^{2}\right)}{{\left(a_{l}b_{r}-b_{l}a_{r}\right)}^{2}+{\left(b_{l}c_{r}-c_{l}b_{r}\right)}^{2}+{\left(a_{l}c_{r}-c_{l}a_{r}\right)}^{2}} \end{array}\right.$$

**Figure 8:**
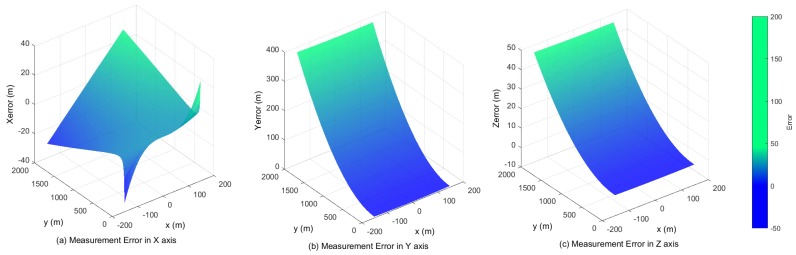
The baseline is 15 m, and the focus is 100 mm. The measurement errors in the *X*, *Z* and *Y* axis with 2000 m.

The angle between the UAV landing trajectory and the runway area is usually between 3° and 7°. By considering 1 mrad normal distributed disturbance (the accuracy of the PTU is 0.006°), Figure 8 illustrates measurement errors of $x_{M}$, $y_{M}$ and $z_{M}$ in the case of different points (x,y)∈S, where S={(x,y)|-50≤x≤50,20≤y≤1000}.

**Figure 9:**
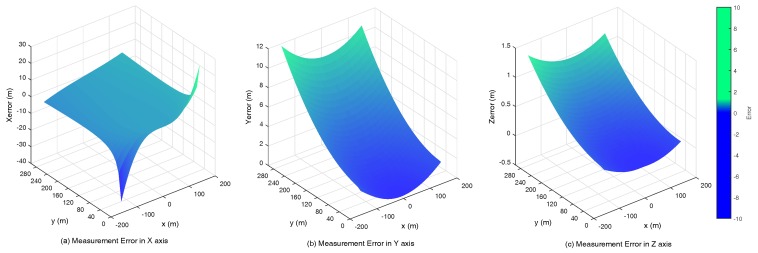
The baseline is 15 m, and the focus is 100 mm. The measurement errors in the *X*, *Z* and *Y* axis with 280 m.

**Figure 10:**
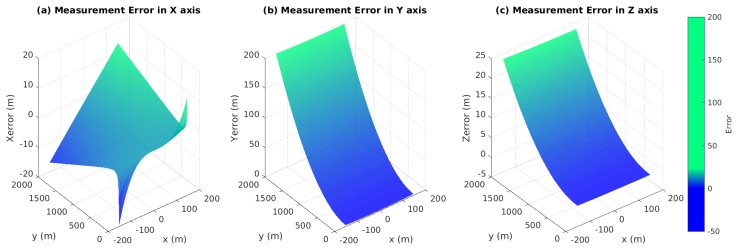
The baseline is 20 m, and the focus is 100 mm. The measurement errors in the *X*, *Z* and *Y* axis with 2000 m.

Obviously, the errors at a considerable distance are notable, but their incidence declines while the aircraft is close to the runway. When the UAV is only 100 m to the landing area, the error of altitude is about 0.02 m, which is dependable for the landing task, as shown in Figure 9. Figure 10 shows that the navigation could be improved at the same distance with a large baseline configuration (20 m).

Furthermore, the errors are much smaller as the UAV lands aligned with the center line of the runway. Table 1 and Table 2 illustrate that the error varies non-linearly and decreases significantly as the target approaches the touch down point. The smaller the disturbance of the system is, the better the accuracy in each axis will be.

Different from the traditional binocular vision system, the optical axes of each vision unit are not parallel during the operation, and there is an initial offset between the camera optical center and the rotation axes of PTU. Therefore, the traditional checkerboard pattern calibration method is not sufficient and convenient to obtain the stereo system parameters for our large baseline system. To solve the calibration issue, we firstly chose the intrinsic camera model, which includes the principal point displacement, optical distortions, skew angle, etc. Each camera should be calibrated separately by the classical black-white chessboard method with the help of the calibration module from OpenCV. Secondly, we setup the setting points with the help of the differential Global Positioning System (DGPS) module and calibrate the system based on the PTU rotation angle, coordinates and the ground-truth position of the setting points.

### 4.3. Rotation Compensation

According to the above discussion, the 3D location estimation depends largely on the precision of the target center position in the image plane. Our previous work [16,17,20] introduced saliency-inspired and the Chan–Vese model method to track the UAV during the landing progress. Both of these approaches predict and extract the center of the UAV position without considering the PTU rotation. However, in the practical situation, the PTU might jump suddenly due to the disturbance of the control signal and the unexpected maneuver of the UAV. We define the target center position as $\left(x_{t},y_{t}\right)$ in the image frame, which can be predicted iteratively by:

(23) $$\begin{array}{c} x_{t}=f\frac{x_{t-1}-f\psi }{x_{t-1}\psi -\phi y_{t-1}+f} \end{array}$$

(24) $$\begin{array}{c} y_{t}=f\frac{y_{t-1}-f\phi }{x_{t-1}\psi -\phi y_{t-1}+f} \end{array}$$

where ψ and ϕ are the PTU rotation angles and *f* is the camera focal length. The precision of the bounding box prediction (BBP) could be improved by the PTU rotation compensation.

### 4.4. Localization Framework

**Figure 11:**
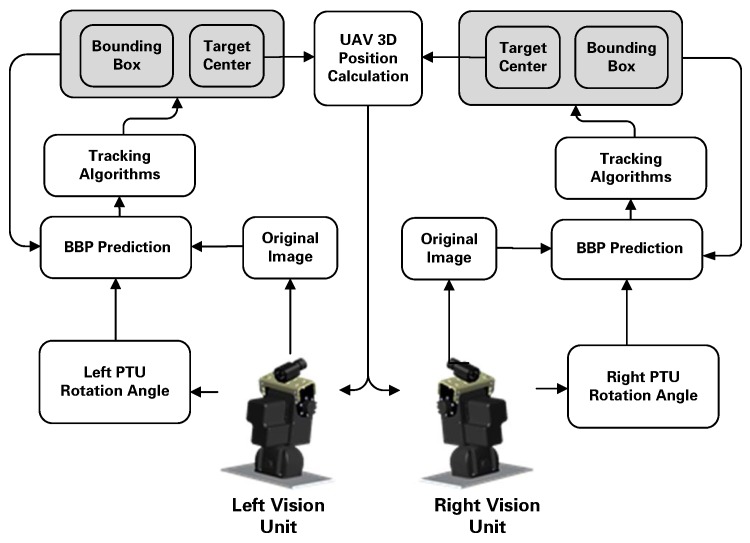
PTU-based UAV localization framework.

In the ensemble configuration, we separate the vehicle guidance and control into an inner loop and an outer loop, because it is a much simpler and well-tested design approach. As the inner loop controller already exists in the autopilot, we developed an efficient and robust outer navigation loop, which manages the visual information with the on-board sensors. Figure 11 presents the separated long baseline stereo localization frame.

## 5. Experiments and Discussion

### 5.1. Experiments’ Setup

For visible light camera, we selected DFK 23G445, which was developed by Imaging Source GmbH. The sensor of this camera is the Sony ICX445AQA equipped with the GigE interface, which has high data transfer rates, typically up to 1000 Mbit/s. This camera has an image resolution of 1280 × 960 with the RGB32 color model and a maximum frame rate of 30 fps. The lens of the vision system we adopted is 100 mm, and the baseline is 10 m. To extend the field of view, we adopted precision PTU to actuate the camera. PTU-D300E is a high performance product from FLIR. Its pan/tilt speeds up to 50°/s with the position resolution of 0.006°. Moreover, it is a user-programmable product integrating Ethernet and RS-232 interface. The real-time command interface supports advanced applications such as video tracking. We set up the camera on the top bracketing, and the assembled individual vision system is illustrated in Figure 3.

This experimental test-bed is a customized fixed-wing aircraft, which is a gasoline-powered radio-controlled model aircraft. The on-board autopilot allowed for the aircraft to perform simple commanded maneuvers. Our autopilot module is iFLY-F1A, and the navigation module is iFLY-G2 [21], which is a small six-DOF (degree of freedom) navigation system. This module supports real-time 3D information including attitude angle, angular rate, position, speed, acceleration, true air speed and calibrated air speed. F1A is connected with G2 through the RS-232 serial port. Table 3 lists the other technical specifications of the UAV platform. Communication is crucial in the landing navigation framework, because the relative localization is broadcast through the radio. The navigation data are sent using an advanced radio modem that transmits and receives on the 900-MHz band. The XTend RF modems support up to 22 km outdoor communication with the interface data rates from 10 bps–23,000 bps, which is sufficient to transfer GNSS data and predicted position from the ground station to the on-board navigation modem.

The landing procedure was divided into four sections: (1) the UAV takeoff from the runway; (2) cruise near the landing area in a large range to test the control system; (3) cruise near the landing area in a small range; and after the UAV is locked by the visual system and received the visual references, the UAV control system was using vision-based localization data, and the GPS data was only recorded as the benchmark; (4) safely landing back on the runway.

### 5.2. Results and Discussion

**Figure 12:**
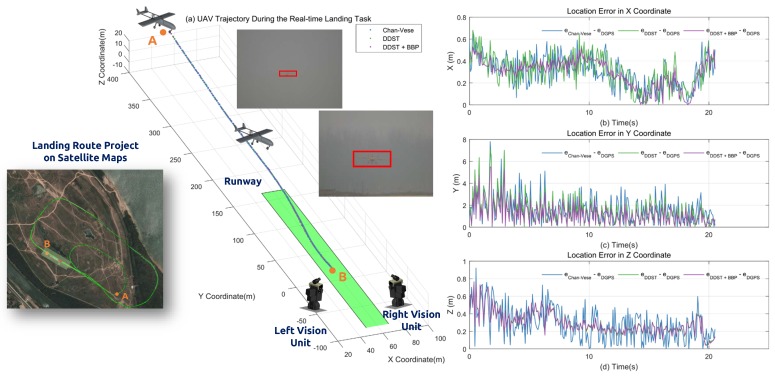
Final approach of the fixed-wing UAV.

Based on the results of the simulation, eight sets of experimental results are conduced to establish the feasibility of the proposed approach. The results are shown in Table 4. Considering the real-time capability and the precision of the target detection, we modified the original discriminative scale space tracker (DSST) [22], which additionally calculates a one-dimensional discriminative scale filter to evaluate the target size. In the realistic application, it is a very critical requirement that the lateral deviation error from the middle line of the runway and the lateral acceleration of the vehicle be perfectly eliminated to minimize the damage of the vehicle. Figure 12 illustrates the approach results. The left image shows the landing waypoints projecting on a satellite map where *A* is the locking point of the ground landing and *B* is the desired touch down point on the runway. In addition, the three 3D landing curves represent the calculated results from the Chan–Vese, DDST and DDST with BBP methods. To compare with the ground truth, recording during the landing process by DGPS, the location errors of each axis are lists on the right side. In *X* and *Z*-axis, the location error decreases while the vehicle approaches the landing area. The error in *Y*-axis has larger error compared with *X* and *Z*-axis, and the disturbance is significant.

As the theoretical and simulation result discussed, the localization errors in each axis are large when the UAV is far way from the ground visual system. To illustrate the result more clearly, we compared the localization results with DGPS at separate intervals, which are shown in Table 5. Previously, the average errors of each axis at a large distance (more than 400 m) are large, especially in the depth dimension. The comparison of the average frame rate of different kinds of tracking algorithms is listed in Table 6. The DSST results have the best real-time performance, which reaches 21.345 fps, and has better accuracy compared with the mean shift method, which has similar process speed. We also calculated the errors in $O_{c}$($i^{O,c}$,$j^{O,c}$, $k^{O,c}$) coordinates by implementing those tracking methods with the identical landing imaging streaming and PTU status. The results are shown at separate interval in Table 7, Table 8 and Table 9. In the accuracy measurement, the DSST with BBP calculates the 3D position more precisely at the cost of a slower frame rate.

## 6. Concluding Remarks

This paper presents a complete localization framework of the ground-based stereo guidance system. This system could be used to pilot the UAV for landing autonomously and safely in the GNSS-denied scenario. Compared with the onboard solutions and other state-of-the-art ground-based approaches, this ground-based system profited enormously from the computation capacity and flexible configuration with the baseline and sensors. The separate deployed configurations did not improve the detection distance, which was discussed in our previous works [15]; however, they enhance the maximum range for depth estimation. Although the system has some pitfalls, such as the low accuracy at a long distance in the depth axis and not supporting the attitude measurement, this low-cost system could be arranged quickly for any proposed environment. Additional future work will focus on estimate errors over time and investigate methods to improve inevitable error propagation through the inclusion of additional sensors, such as GNSS and on-board sensors.

## Figures and Tables

**Table 1:** Errors when the center extraction of the aircraft with 1-pixel disturbance, *f* = 100 mm and *D* = 10 m.

Error (m)/Distance (m)	4000	3000	2000	1000	500	200	100	50
Xerror	−1.44	−1.16	−0.63	−0.65	−0.25	−0.10	−0.05	−0.05
Yerror	1141.92	692.31	333.44	195.65	23.82	3.92	1.00	0.25
Zerror	133.42	82.62	39.24	22.70	2.43	0.28	0.03	−0.02

**Table 2:** Errors when the center extraction of the aircraft with 5-pixel disturbance, *f* = 100 mm and *D* = 10 m.

Error (m)/Distance (m)	4000	3000	2000	1000	500	200	100	50
Xerror	−3.33	−3.00	−2.53	−2.14	−1.02	−0.46	−0.23	−0.13
Yerror	2663.28	1800.31	1000.11	642.87	100.23	18.19	4.17	1.23
Zerror	320.66	214.93	117.73	74.57	10.24	1.32	0.09	−0.07

**Table 3:** The technical specifications of Pioneer.

Items	Description
Vehicle mass	9000 g
Maximum Payload mass	5000 g
Diameter	2900 mm
Flight duration	up to 180 min
Cruising speed	30.0 m/s

**Table 4:** Eight experiment results in different weather condition.

No.	Weather Condition	Detection Distance	RMSE $i^{O,c}$ (m)	RMSE $j^{O,c}$ (m)	RMSE $k^{O,c}$ (m)
**1**	Clear	848.735	0.330	1.465	0.291
**2**	Clear	**892.134**	0.241	1.292	**0.229**
**3**	Clear	872.311	0.389	1.322	0.293
**4**	Clear	847.373	0.261	1.413	0.245
**5**	Clear	857.117	**0.251**	**1.252**	0.312
**6**	Overcast	491.193	0.503	1.689	0.602
**7**	Overcast	503.175	0.495	**1.353**	**0.587**
**8**	Overcast	**534.238**	**0.482**	1.781	0.592

**Table 5:** Localization error in each axis at separated intervals with the DSST and bounding box prediction (BBP) algorithms.

Interval (m)	$i^{O,c}$ (m)	$j^{O,c}$ (m)	$k^{O,c}$ (m)	Interval (m)	$i^{O,c}$ (m)	$j^{O,c}$ (m)	$k^{O,c}$ (m)
**600∼580**	0.348	1.862	0.441	**300∼280**	0.138	1.526	0.153
**580∼560**	0.322	1.444	0.362	**280∼260**	0.142	1.413	0.178
**560∼540**	0.218	1.557	0.327	**260∼240**	0.103	1.114	0.124
**540∼520**	0.197	1.558	0.284	**240∼220**	0.094	0.711	0.132
**520∼500**	0.228	1.841	0.183	**220∼200**	0.105	0.898	0.143
**500∼480**	0.229	1.430	0.226	**200∼180**	0.151	0.831	0.163
**480∼460**	0.192	1.483	0.233	**180∼160**	0.163	0.842	0.134
**460∼440**	0.183	1.472	0.239	**160∼140**	0.157	0.913	0.192
**440∼420**	0.192	1.431	0.121	**140∼120**	0.142	0.725	0.160
**420∼400**	0.191	1.663	0.199	**120∼100**	0.169	0.922	0.149
**400∼380**	0.169	1.662	0.193	**100∼80**	0.147	0.797	0.069
**380∼360**	0.171	1.542	0.185	**80∼60**	0.133	0.697	0.079
**360∼340**	0.173	1.541	0.183	**60∼40**	0.114	0.441	0.068
**340∼320**	0.153	1.333	0.161	**40∼20**	0.124	0.312	0.064
**320∼300**	0.156	1.311	0.163	**20∼00**	0.082	0.284	0.103

**Table 6:** Average frame rate of the tracking algorithms.

	Previous Methods				Our Methods	
	Mean Shift [23]	AdaBoost [24]	Chan–Vese [25]	Saliency-Inspired [26]	DSST	DSST + BBP
**Average Frame Rate (fps)**	20.131	13.152	7.131	8.013	**28.335**	27.867

**Table 7:** Errors (m) in the $i^{O,c}$-axis with tracking algorithms at separate intervals (m).

Interval	Previous Methods				Proposed Methods	
	Mean Shift [23]	AdaBoost [24]	Chan–Vese [25]	Saliency-Inspired [26]	DSST	DSST + BBP
500–400	1.113	0.911	1.201	1.181	0.034	0.332
400–300	0.932	0.891	0.921	0.943	0.029	0.027
300–200	0.562	0.416	0.713	0.765	0.017	0.016
200–100	0.329	0.287	0.512	0.447	0.013	0.013
<100	0.213	0.120	0.341	0.281	0.013	0.011

**Table 8:** Errors (m) in the $j^{O,c}$-axis with tracking algorithms at separate intervals (m).

Interval	Previous Methods				Proposed Methods	
	Mean Shift [23]	AdaBoost [24]	Chan–Vese [25]	Saliency-Inspired [26]	DSST	DSST + BBP
500–400	11.923	6.238	3.661	3.231	2.218	1.761
400–300	7.317	5.721	2.887	2.902	1.876	1.632
300–200	5.365	2.576	1.799	1.767	1.703	1.371
200–100	3.897	1.739	1.310	1.134	0.981	1.131
<100	1.762	0.780	0.737	0.692	0.763	0.541

**Table 9:** Errors (m) in the $k^{O,c}$-axis with tracking algorithms at separate intervals (m).

Interval	Previous Methods				Proposed Methods	
	Mean Shift [23]	AdaBoost [24]	Chan–Vese [25]	Saliency-Inspired [26]	DSST	DSST + BBP
500–400	1.231	1.087	1.387	1.299	0.487	0.484
400–300	0.976	0.901	0.762	0.876	0.370	0.325
300–200	0.812	0.557	0.337	0.612	0.459	0.313
200–100	0.438	0.312	0.489	0.532	0.141	0.179
<100	0.301	0.211	0.401	0.321	0.102	0.103
